# The Diagnosis of Aneurism

**Published:** 1895-10-26

**Authors:** A. Pearce Gould

**Affiliations:** Senior Assistant Surgeon to the Middlesex Hospital.


### The Diagnosis of Aneurism.

By A. Pearce Gould, F.R.C.S., Senior Assistant Surgeon to the Middlesex Hospital.

In my last article I described the symptoms to which aneurism may give rise, and must now pass on to discuss the diagnostic importance and value of these various phenomena. Recalling my definition of an aneurism—a blood-tumour communicating with an artery—it is evident that the diagnosis of this condition must depend upon the recognition (1) of a tumour, and (2) of the communication of that tumour with the lumen of an artery. It is important to lay stress upon the first of these points, however self-evident it may appear to be. Many mistakes have arisen from its neglect. The recognition of an abnormal pulsation is not sufficient to justify the diagnosis of aneurism, but in every case the surgeon must first determine whether there is or is not a tumour which communicates with an artery. A "pulsating aorta" is often diagnosed as an aneurism by an untrained observer; the pulsation is oftentimes very distinct, and there are certain local signs of pain and dyspepsia which can easily be attributed to the pressure of a tumour growing behind the stomach. In such cases careful palpation will at once determine that there is no tumour nor any enlargement of the aorta, and this will immediately exclude under aneurism. If the surgeon is doubtful whether there is a tumour or not, a very simple way of determining it is to place the patient in the kneeling position and with belly quite lax. If, now, on placing the hand very lightly on the abdomen and keeping the belly muscles relaxed, the surgeon feels distinct pulsation, the probability is very great that there is an aneurism of one of the high abdominal arteries; if no pulsation is felt when the viscera are allowed to fall from the spine to the aorta, it cannot be caused by a tumour, and a tumour is a prime requisite in the diagnosis. Another case in which error arises is where the pulsation of an abnormally placed artery is attributed to an aneurism. Again, in very thin people the normal arteries may be mistaken for aneurisms, particularly at the root of the neck and the groin; and if these arteries are the seat of atheroma, making them stand out more prominently, or if they have yielded in their length and become a little tortuous, the mistake in diagnosis is still more liable to occur. These errors may all be obviated by noticing the absence of a distinct tumour. In many cases of intrathoracic aneurism the presence of a tumour is recognised, not by the sense of touch, but by the signs of pressure upon and displacement of certain of the viscera, particularly the lungs. It is as true as regards the cranium and thorax as it is of a limb, that the first step in the diagnosis of an aneurism must always be to establish the existence of a tumour.

Of equal importance is it to show that such a tumour communicates with the lumen of an artery. An aneurismal tumour is always over and fixed to an artery. By no manipulation can it be detached from or in any way separated from an artery. The special features of the pulsation in an aneurism are the signs of greatest importance in the diagnosis of an aneurism. It is important to remember that every pulsating tumour is not an aneurism: the pulsation must be of such a kind as to show that the tumour is actually filled out by blood passing into it from the artery at each beat of the heart. The pulsation is expansile. This is best recognised by placing the fingers of the two hands at opposide sides of the tumour and noticing that they are thrust apart with each heart beat. This expansion of the tumour is felt in every part of it, and a tumour which only pulsates at certain spots is almost certainly not an aneurism. Along with this great sign of aneurism must be placed the shrinking of the tumour when the artery above is compressed. The one shows that the blood flows out of the artery into the tumour, the other that when the blood pressure in the artery is greatly lowered blood flows into it from the aneurism. This sign must be elicited with care. With one hand the artery is compressed well on the cardiac side of the tumour; this at once arrests the pulsation in the tumour (a point of no importance), and the observer's other hand gently laid upon it detects a slight shrinking of the tumour; then, by gently compressing it with the hand, the sac is felt to shrink still more. The rapidity with which this shrinking of the tumour from emptying of the sac into the blood-stream occurs, depends directly upon the size of the mouth of the aneurism, and the degree to which it takes place is in inverse proportion to the amount of clot in the sac. Any case which presents these three signs unmistakably is an aneurism. A tumour which is the seat of expansile pulsation, and which empties a part of its contents into the artery when the direct flow of blood through the vessel is cut off, must be an aneurism.

All the other signs and symptoms of aneurism are of quite secondary importance for the purposes of diagnosis. The bruit may be absent altogether, or it may present the most varied characters; the most useful fact about it is that it is heard of equal intensity all over the aneurismal tumour. The change in the pulse in the arteries beyond the aneurism is usually characteristic.

The thrill is of still less value. The pressure signs are of great clinical moment, and they may be regarded as of some diagnostic value, for they bear witness to the exact relations of the tumour to the structures pressed upon, and by their intensity show the great pressure that is exerted by the tumour, and as the pressure in a large aneurism is much greater than that of any other kind of tumour, the intense pressure signs become of diagnostic value.

In the great majority of cases of external aneurism there is no difficulty in arriving at a correct diagnosis; the same may also be said for any cases of internal aneurism, but there are cases of both forms of the disease which present very great difficulties. The secret of reading these cases aright is for the surgeon to fix his mind on the two cardinal points to which I have referred to-day; he must satisfy himself first that there is a tumour, and then that this tumour communicates with an artery.

In my next article I will discuss cases of pulsating tumours which are not aneurisms, and also of aneurisms which do not pulsate; and then deal with the diagnosis of the various forms of aneurism so far as we are capable of recognising them by their clinical phenomena.

